# Prognostic Factors Associated With Survival in Patients With Diffuse Astrocytoma

**DOI:** 10.3389/fsurg.2021.712350

**Published:** 2021-10-15

**Authors:** Shuo Liu, Xiaoqiang Liu, Weiduan Zhuang

**Affiliations:** Neurology Department, The First Affiliated Hospital of Shantou University Medical College, Shantou, China

**Keywords:** diffuse astrocytoma, epidemiology, prognosis, SEER database, nomogram

## Abstract

**Background:** Diffuse astrocytoma (DA) is a rare disease with inadequately understood epidemiological characteristics and prognosis. Identification of the factors associated with the survival in DA patients is therefore necessary. In this study, we aim to investigate the clinicopathological characteristics of DA to delineate factors influencing the survival of DA.

**Methods:** A population-based cohort study was conducted, utilizing prospectively extracted data from the Surveillance, Epidemiology and End Results (SEER) database. Patients with histological diagnosis of DA in the SEER database from 1973 to 2017 were included.

**Results:** A total of 799 participants with DA were included, consisting of 95.9% fibrillary astrocytoma and 4.1% protoplasmic variants. The average age of participants was 41.9 years, with 57.2% being male. The majority of the population was white (87.5%). More than half (53.9%) of the patients were married. DA arose mostly in the cerebrum (63.8%). Around 71.6% of the population had received surgical treatment. The overall 1-, 3-, 5-, and 10-year survival rate were 73.7, 55.2, 49.4, and 37.6%, respectively. Kaplan–Meier analysis showed that age at diagnosis, marital status, primary tumor site, tumor size, and surgery was possibly associated with cancer-specific survival (CSS) (*p* < 0.05). Multivariate Cox proportional hazard analysis indicated that surgery was a protective factor whereas older age, larger tumor size, and tumor in the brainstem were harmful factors for patients with DA. Moreover, a nomogram predicting 5- and 10-year survival probability for DA was developed.

**Conclusions:** Age, primary tumor site, tumor size, and surgery were associated with the survival of patients with DA.

## Introduction

Astrocytomas account for about 80% of adult gliomas. They are the most common gliomas in the fourth through the sixth decades of life. On the basis of histological features, astrocytomas are stratified into pilocytic astrocytoma (grade I), diffuse astrocytoma (DA) (grade II), anaplastic astrocytoma (grade III), and glioblastoma (grade IV) by the World Health Organization (WHO) (1). DA is an infiltrating, hypercellular tumor composed of atypical cells that show astrocytic differentiation and mildly increased mitotic activity (2). It comprises 10–15% of all astrocytic brain tumors (3). Although DA is defined as a WHO grade II tumor, it regularly undergoes a malignant transformation into anaplastic astrocytoma and glioblastoma, eventually resulting in death (4). DA consists of fibrillary astrocytoma, protoplasmic astrocytoma, and gemistocytic astrocytoma. Due to the rapid transformation into a higher grade (5), gemistocytic astrocytoma is considered a variant of anaplastic astrocytoma by some experts (6).

Clinical symptoms of DA vary depending on the location of the tumor. Seizures, headaches, and focal neurologic deficits are the most frequent presenting symptoms. In comparison with other WHO grade II tumors, DA has a relatively worse prognosis (7). On the basis of the United States national cancer registries (1995–2009), the 5-year overall survival (OS) rate of DA is 47.1% (8). In earlier studies, age is proved as a prognostic factor for survival in DA (9, 10). However, studies about clinicopathological characteristics of DA are scarce in the literature at present. Factors influencing the prognosis of DA are also unclear.

Therefore, we conducted an analysis of DA through the population-based SEER database. In this study, we aim to investigate the clinicopathological characteristics of DA to delineate factors influencing the survival of DA.

## Methods

### Patient Population

The SEER program is a cancer registry that prospectively collects information of the patients, including clinicopathological characteristics of cancer and survival. It is supported by the National Cancer Institute. Our study was designed as a population-based retrospective study. We used the latest release data of the SEER database, which constituted of patients documented from the years 1973–2017. According to the International Classification of Diseases for Oncology, Third Edition (ICD-O-3), patients diagnosed with DA as their primary tumor were identified. The ICD-O-3 morphology codes were 9,410 (protoplasmic astrocytoma) and 9,420 (fibrillary astrocytoma). Demographic features of these patients and clinicopathological characteristics of DA were collected.

### Definition of Variables

The age of the patient at diagnosis, race, sex, marital status, primary tumor site, histological type, tumor size, surgical treatment, survival duration in months, and survival status were collected in this study. Patients with unclear information on any of the collected variables were excluded. The race was grouped into three categories: white, black, and others (American Indian/Alaska Native, Asian, or Pacific Islander). Similarly, marital status at diagnosis was classified as single, married, and others (separated/divorced/widowed) (Sep/Div/Wid). The primary tumor site was recategorized into five distinct categories including cerebrum (C71.1/C71.2/C71.3/C71.4/C71.5), cerebellum (C71.6), brainstem (C71.7), spinal cord (C72.0), and others. The primary outcome was cancer-specific survival (CSS).

### Statistical Analysis

Cancer-specific survival stratified by each factor was delineated by a Kaplan–Meier curve. The connection between clinicopathological factors and CSS was analyzed using Cox proportional hazards model. Statistically significant variables in univariate Cox analysis were further included in multivariate Cox analysis. For each patient, significant prognostic factors were further put into the nomogram calculator to get a predicted survival rate at 5- and 10 years. The C-index and receiver operating characteristic (ROC) curve were utilized to evaluate the accuracy of the nomogram. The C-index and area under the curve (AUC) ranged from 0.5 to 1. A Higher C-index value or AUC indicated a better prognostic model. A two-tailed *P* ≤ 0.05 was determined as statistically significant. R software (version 3.5.0) was utilized to perform the statistical analysis.

## Results

### Incidence

A total of 799 patients with DA(mean age 41.9 years) were included (Table 1), consisting of 766 fibrillary astrocytoma (95.9%) and 33 protoplasmic variants (4.1%). Among this population, 457 were male (57.2%). The majority of the population was white (87.5%). Most subjects were married (53.9%), whereas 33.9% were single. Of these patients, DA arose mostly in the cerebrum (63.8%), while to a lesser extent in the cerebellum (4.8%), brainstem (3.5%), and spinal cord (1.8%). With respect to tumor characteristics, the mean tumor size was 41.1 mm. Around 71.6% of the population had surgery to decrease the tumor burden. Among all the cases, DA accounted for 436 deaths (54.6%).

**Table 1:** Characteristics of patients with diffuse astrocytoma.

**Characteristics**		**Number**
Number		799
Age (year) ± SD		41.9 ± 20.9
Sex	Male	457 (57.2%)
	Female	342 (42.8%)
Race	White	699 (87.5%)
	Black	56 (7.0%)
	Others	44 (5.5%)
Marital status	Single	271 (33.9%)
	Married	431 (53.9%)
	Others	97 (12.2%)
Primary site	Brainstem	28 (3.5%)
	Cerebrum	510 (63.8%)
	Cerebellum	38 (4.8%)
	Spinal cord	14 (1.8%)
	Others	209 (26.1%)
Histology	Protoplasmic	33 (4.1%)
	Fibrillary	766 (95.9%)
Tumor size (mm)	41.1 ± 19.8
Surgery	Yes	572 (71.6%)
	No	227 (28.4%)
Status	Alive	326 (40.8%)
	Dead	473 (59.2%)

### Survival

**Figure 1:**
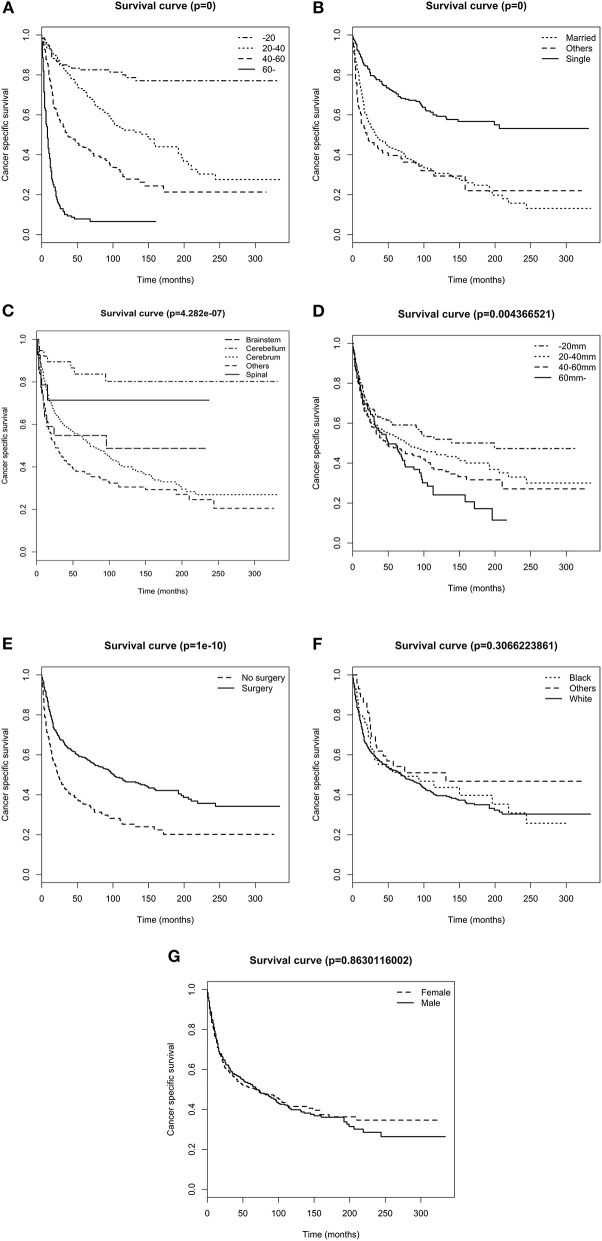
Survival analysis of patients stratified by risk factors. **(A)** age **(B)** marital status, **(C)** primary tumor site, **(D)** tumor size, **(E)** surgery, **(F)** race, and **(G)** sex.

The overall 1-, 3-,5-, and 10-year survival rates were 73.7, 55.2, 49.4, and 37.6%, respectively. The cancer specific 1-, 3-, 5-, and 10-year survival rates were 75.2, 57.3, 51.8, and 40.5%, respectively. The Kaplan–Meier analysis showed that age at diagnosis (Figure 1A), marital status (Figure 1B), primary tumor site, (Figure 1C), tumor size (Figure 1D), and surgery (Figure 1E) were possibly associated with CSS, whereas race (Figure 1F) and sex (Figure 1G) were not associated with CSS. All included variables were admitted into the univariate Cox analysis. As shown in Table 2, the results showed that age, marital status, primary tumor site, tumor size, and surgery each had statistically significant association with CSS (*p* < 0.05). No significant difference was noticed across sex and race. Significant variables in the univariate Cox analysis were further included in the multivariate Cox analysis, and the result demonstrated that age, primary tumor site, tumor size, and surgery were independent prognostic factors for DA. Surgery was a protective factor whereas older age, larger tumor size, and tumor in the brainstem were harmful factors for patients with DA.

**Table 2:** Univariate and multivariate Cox proportional hazard analyses of clinical characteristics for cancer-specific survival rates.

**Factor**	**Category**	**Univariate**		**Multivariate**	
		**HR (95% CI)**	***P*-value**	**HR (95% CI)**	***P*-value**
Age		1.052 (1.047–1.058)	<2e^−16^	1.052 (1.046–1.059)	<2e^−16^
Sex	Female	Reference			
	Male	1.017 (0.841–1.23)	0.862	–	–
Race	Black	Reference			
	White	1.061 (0.743–1.515)	0.745	–	–
	Others	0.755 (0.437–1.304)	0.313	–	–
Marital status	Married	Reference			
	Single	0.398 (0.316–0.502)	5.64e^−15^	1.045 (0.815–1.340)	0.731
	Separated/Divorced/Widowed	1.150 (0.871–1.518)	0.325	0.897 (0.674–1.194)	0.455
Primary site	Brainstem	Reference			
	Cerebral	1.113 (0.638–1.942)	0.704	0.387 (0.216–0.695)	0.001
	Cerebellum	0.257 (0.103–0.645)	0.004	0.235 (0.093–0.597)	0.002
	Spinal cord	0.581 (0.190–1.783)	0.343	0.389 (0.125–1.208)	0.102
	Others	1.579 (0.893–2.789)	0.116	0.538 (0.296–0.980)	0.042
Tumor size		1.010 (1.005–1.014)	3.37e^−5^	1.009 (1.005–1.014)	0.0001
Surgery	No surgery	Reference			
	Surgery	0.527 (0.433–0.642)	2.07e^−10^	0.699 (0.569–0.859)	0.0007

### Nomogram

**Figure 2:**
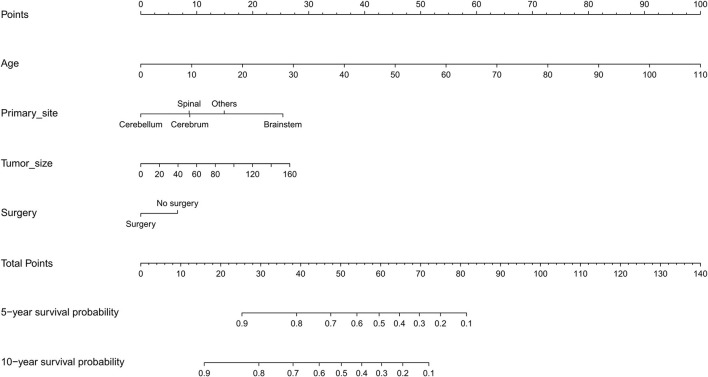
Nomogram for predicting 5- and 10-year survival probability of diffuse astrocytoma.

**Figure 3:**
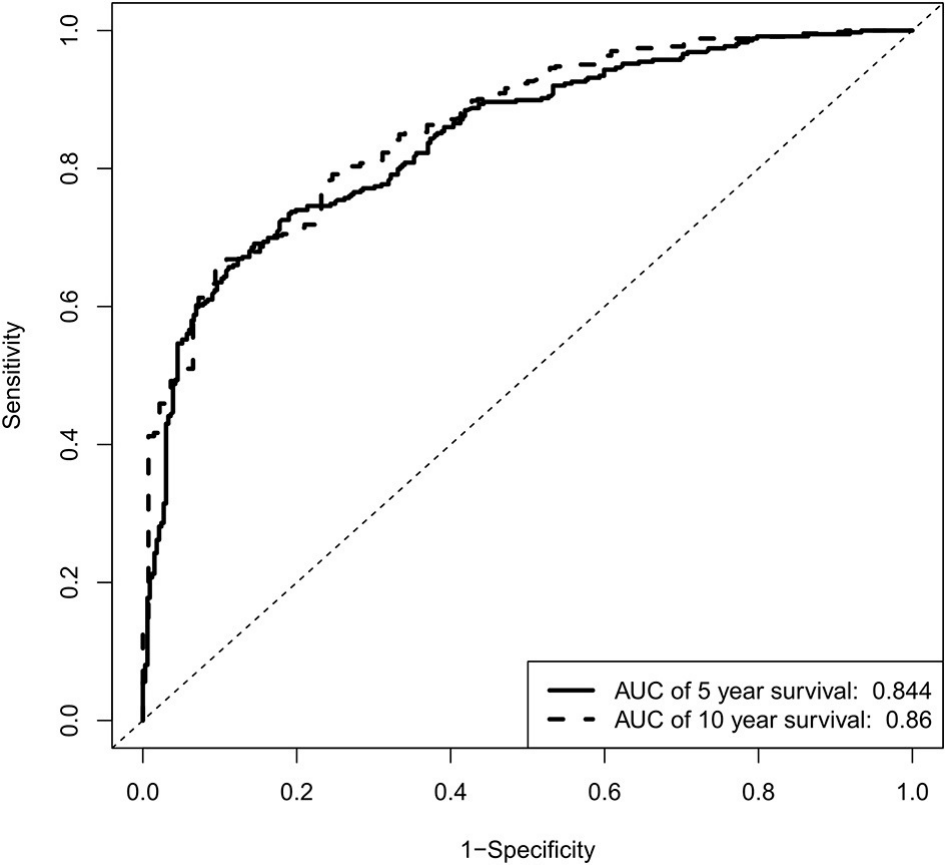
ROC curves for validating nomogram model.

**Figure 4:**
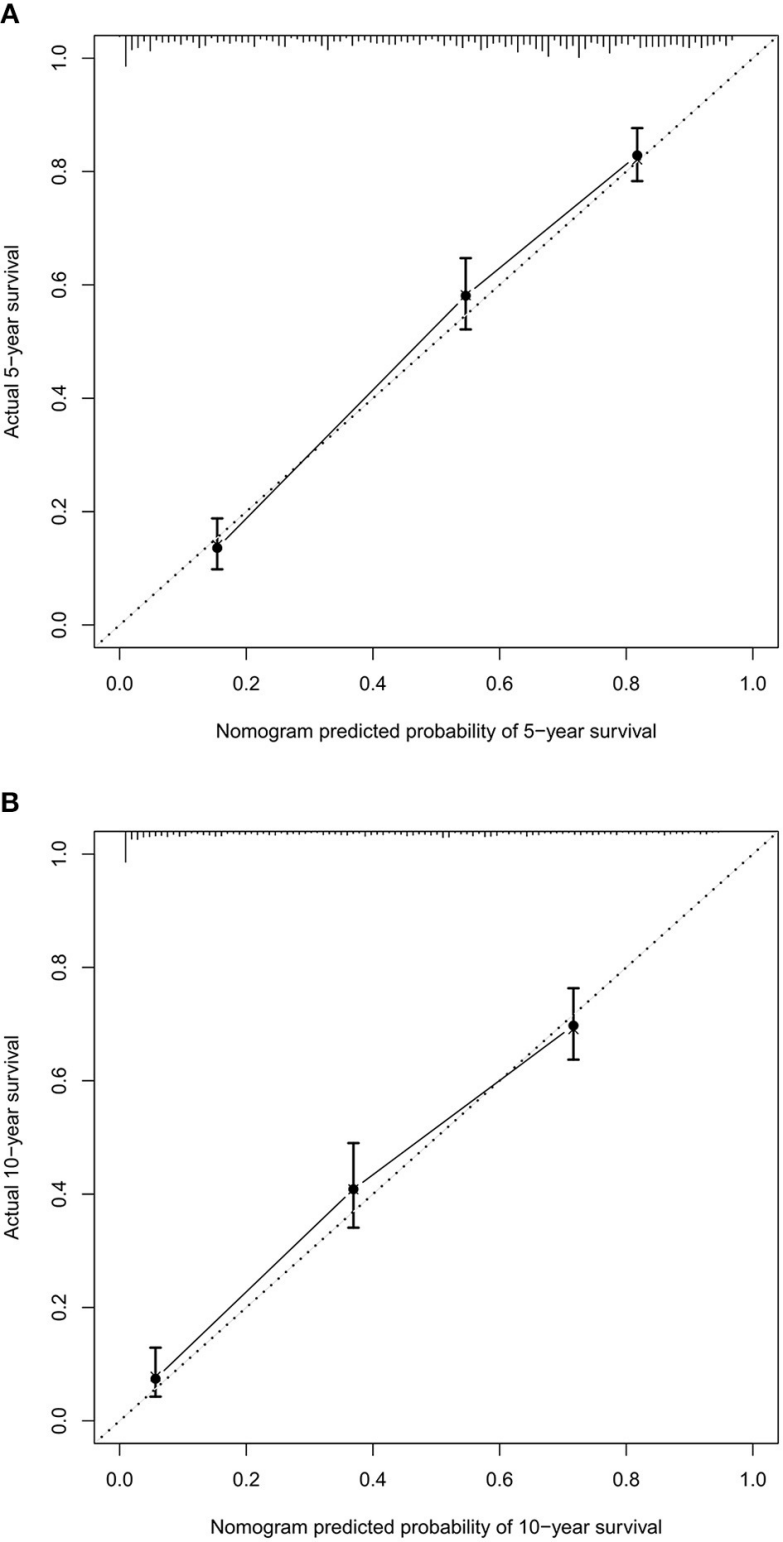
The calibration curve for predicting patient survival at 5- **(A)** and 10-years **(B)**. Nomogram predicted probability of cancer-specific survival (CSS) is plotted on the *x*-axis, actual CSS is plotted on the *y*-axis.

Based on the independent predictors from the multivariate Cox proportional hazards analysis, a nomogram predicting 5- and 10-year CSS for each of the DA predictors was constructed (Figure 2). This nomogram showed the probability of involvement of each predictor, and higher points correlated with lower survival probability. It also revealed that age contributed most to the prognosis, followed by tumor size, primary site, and surgery. By adding the scores of each predictor, the CSS probability of each patient with DA can be calculated. An interesting observation was that DA in the brainstem led to the lowest survival probability compared with other sites. Likewise, older patients and larger tumor sizes would appear to indicate a lower survival probability. The C-index of the nomogram was 0.774. In the plotted ROC curves, the AUC was 0.844 and 0.860 at 5- and 10-years, indicating a moderate accuracy in our nomogram model (Figure 3). Moreover, an optimal agreement between actual observation and the nomogram prediction was seen in the calibration plot for the probability of survival at 5- or 10-years (Figures 4A,B).

## Discussion

Reports are scarce regarding the detailed clinicopathological characteristics of DA. Most present studies of DA are small case series. Therefore, epidemiological characteristics and factors influencing the prognosis of DA remain unclear. To the best of our knowledge, our study is the largest one to depict clinicopathological characteristics of DA and describe factors influencing its survival.

Diffuse astrocytoma has been previously reported in younger people. In our study, the average age at diagnosis was 41.9 years. The literature suggested that the mean age at diagnosis of DA patients was 45.7 years, which was slightly older than our subjects (11). This disease mostly occurs in young people, which increases the economic and social burden for the entire family and society. Our data demonstrated that the white was predominately affected and over half of the patients were married. We also found that DA most commonly arose in the cerebrum. The previous study had reported that DA was more prevalent among men (51.7:48.3 male: female) (12) and our male:female (57.2:42.8) ratio was slightly higher when compared with literature. In literature, protoplasmic astrocytoma was a rare variant and fibrillary type was the most common, representing more than 85% of all DA (13). Our data showed that only 4.1% were protoplasmic astrocytoma whereas 95.9% were fibrillary astrocytoma. The previous study had reported that the 5- and 10-year survival rates for DA were 48 and 36%, respectively (14). In our study, 49.4% lived more than 5 years whereas 37.6% lived more than 10 years.

Among the clinical variables, age was identified as a significant prognostic factor for the survival of DA in our study. Older patients had a much poorer prognosis. This result was in line with the literature (10, 15–18). A previous study had put forward the definite association between age and survival of DA, with a worse prognosis in older patients (9). Moreover, age values of 47 and 63 years were calculated as two optimal cutoff values to distinguish good, moderate, and poor survival.

Surprisingly, we observed that tumor size affected the survival of DA. Similarly, the tumor size value of 25 and 44 mm were two optimal cutoff values to distinguish good, moderate, and poor survival. We were also the first to report the effect of primary tumor site on the survival of DA. DA in the brainstem had the worst survival rate compared with those in other sites. A possible interpretation is that the brainstem plays an important role in the regulation of cardiac and respiratory function. Tumor in the brainstem is more likely to affect heart rate and breathing than tumors in other sites and it causes a lower survival rate.

Evidence concerning the effect of surgery in DA is rare in literature. Our study revealed that surgery was associated with better survival in DA. However, the surgical types of most included cases were not specified in the database. Therefore, we were unable to further analyze the association between surgical types and survival. Previous research demonstrated that extent of tumor resection was not correlated with patient survival in DA (3). Due to the scarce evidence in DA, we used evidence in low grade glioma (LGG) for reference. In the last few decades, the value of surgery in LGG was controversial. However, recent viewpoints have suggested that biopsy is considered harmful, whereas extensive resection is correlated with a more favorable prognosis (2, 19). Some new concepts also have put forward a new standpoint that supratotal resection is a protective factor relating to the management of LGG (20, 21). However, these conclusions come from studies of LGG, which contain not only DA but also oligodendroglioma and oligoastrocytoma. More studies are needed to prove the value of surgery in DA.

A nomogram is an essential tool of modern medical decision-making (22). It is a graphical demonstration of a statistical prediction model generating survival probability of a specific outcome (23, 24). Doctors could easily figure out the prognosis for patients by using a nomogram of an efficient prognostic system. This could also assist in patient counseling and individualized treatment. Additionally, nomograms are specifically worthy for clinicians to solve complex diseases where no definite clinical guidelines exist. Therefore, we constructed a nomogram predicting CCS of DA based on a large population from the SEER database. The nomogram performed well in predicting survival probability, supported by the C-indexes (0.774) and the ROC curves (AUC of 0.844 and 0.860 at 5- and 10-years, respectively). Prediction of prognosis of patients by utilizing nomograms is straightforward. Firstly, each variable corresponds to relevant “points” in the nomogram by drawing a vertical line. Secondly, “total points” is obtained by summing up all the “points” of each variate. Finally, a vertical line from “total points” to the “survival probability” is drawn to get the corresponding survival probability. For example, consider a 22-year-old (20 points) DA patient, with tumor location of the brainstem (28 points), tumor size of 20 mm (4 points), and received surgery (0 points). After using our nomogram, the “total points” of this patient are 52. The 5- and 10-year CCS probabilities of this patient are about 63 and 48%, respectively.

Our study had several limitations. Firstly, DA is currently defined by both histologic and molecular characteristics, whereas the SEER database does not contain the relevant molecular information (25). However, due to the high degree of accordance (about 80%) between the histologic diagnosis and the molecular diagnosis of glioma, this limitation may be alleviated (26, 27). Secondly, we lacked information about radiotherapy, which may play an important part in survival in DA patients. Ultimately, missing data and selection bias were inevitable because the study design was retrospective.

## Conclusions

In conclusion, our study is the largest one to date to investigate the clinicopathological characteristics and survival for patients with DA. We found that age, primary tumor site, tumor size, and surgery were associated with the survival of patients with DA. These outcomes may contribute to future management in DA patients.

## Data Availability Statement

The datasets presented in this study can be found in online repositories. The names of the repository/repositories and accession number(s) can be found in the article/supplementary material.

## Author Contributions

WZ: conceptualization. SL: formal analysis, project administration, and software. SL, XL, and WZ: methodology. WZ: supervision. SL and XL: writing—original draft. SL and WZ: writing—review and editing. All authors contributed to the article and approved the submitted version.

## Conflict of Interest

The authors declare that the research was conducted in the absence of any commercial or financial relationships that could be construed as a potential conflict of interest.

## Publisher's Note

All claims expressed in this article are solely those of the authors and do not necessarily represent those of their affiliated organizations, or those of the publisher, the editors and the reviewers. Any product that may be evaluated in this article, or claim that may be made by its manufacturer, is not guaranteed or endorsed by the publisher.
